# Predictive value of protease‐activated receptor‐2 (PAR_2_) in cervical cancer metastasis

**DOI:** 10.1111/jcmm.16227

**Published:** 2020-12-23

**Authors:** Shengnan He, Meiquan Xu, Zhen Xiong, Ye Hu, Qin Huo, Jingxiao Lu, Yuntao Lin, Lan Yang

**Affiliations:** ^1^ Biobank of Shenzhen Second People's Hospital Health Science Center Shenzhen Second People's Hospital The First Affiliated Hospital of Shenzhen University Shenzhen China; ^2^ Pathology Department of Shenzhen Second People's Hospital Health Science Center Shenzhen Second People's Hospital The First Affiliated Hospital of Shenzhen University Shenzhen China; ^3^ Wuhan Children’s Hospital Tongji Medical College Huazhong University of Science & Technology Wuhan China; ^4^ Department of Graduate School University of South China Hengyang China; ^5^ Department of Oral and Maxillofacial Surgery Peking University Shenzhen Hospital Shenzhen China; ^6^ Department of Gastroenterology of Shenzhen Second People's Hospital Health Science Center Shenzhen Second People's Hospital The First Affiliated Hospital of Shenzhen University Shenzhen China

**Keywords:** cervical squamous cell carcinoma and endocervical adenocarcinoma (CESC), Grb‐associated binding protein 2 (Gab2), metastasis, miR‐125b, protease‐activated receptor 2 (PAR_2_)

## Abstract

Metastasis is the primary cause of an unfavourable prognosis in patients with malignant cancer. Over the last decade, the role of proteinases in the tumour microenvironment has attracted increasing attention. As a sensor of proteinases, proteinase‐activated receptor 2 (PAR_2_) plays crucial roles in the metastatic progression of cervical cancer. In the present study, the expression of PAR_2_ in multiple types of cancer was analysed by Gene Expression Profiling Interactive Analysis (GEPIA). Kaplan‐Meier plotter was used to calculate the correlation between survival and the levels of PAR_2_, Grb‐associated binding protein 2(Gab2) and miR‐125b. Immunohistochemistry (IHC) was performed to examine PAR_2_ expression in a tissue microarray (TMA) of CESCs. Empower Stats was used to assess the predictive value of PAR_2_ in the metastatic potential of CESC. We found that PAR_2_ up‐regulation was observed in multiple types of cancer. Moreover, PAR_2_ expression was positively correlated with the clinicopathologic characteristics of CESC. miR‐125b and its target Gab2, which are strongly associated with PAR_2_‐induced cell migration, are well‐characterized as predictors of the prognostic value of CESC. Most importantly, the Cancer Genome Atlas (TCGA) data set analysis showed that the area under the curve (AUC) of the PAR_2_ model was significantly greater than that of the traditional model (0.833 vs 0.790, *P* < .05), demonstrating the predictive value of PAR_2_ in CESC metastasis. Our results suggest that PAR_2_ may serve as a prognostic factor for metastasis in CESC patients.

## 1 INTRODUCTION

Metastasis is not only the main characteristic of malignancy but also the main factor that affects the therapeutic effect and prognosis of patients. In addition to the genetic background of cancer cells, alterations in the microenvironment have emerged as an important factor in regulating the metastatic progression of cancer. In the past, a great deal of research has focused on the microenvironment that surrounds cells and its role in tumour metastasis.^1^ The microenvironment of the tumour invasion front may enable the cancer cells to gain anomalously high motility and penetrate the surrounding stroma.^2^ It is worth noting that the invasion front of a tumour is particularly rich in a variety of proteinases, which facilitate cancer invasion and metastasis by remodelling the extracellular matrix and promote cell migration.^3^

Protease‐activated receptors (PARs) are a subgroup of G protein‐coupled receptors (GPCRs). To date, four members of the PAR family have been discovered: PAR_1_, PAR_3_ and PAR_4_, which are activated by thrombin, and PAR_2,_ which is activated by trypsin, tissue factor, neutrophil elastase and other factors.^3^, ^4^ Previous evidence has shown that PAR_2_ plays an important role in promoting the metastasis of colon cancer cells.^5^, ^6^, ^7^ As the sensor of protease, PAR_2_ and its activating proteinases are typically observed in the invading frontier cells of cancer,^8^ and its expression level is tightly correlated with the switching of a primary tumour from local to metastatic spread.^7^, ^9^, ^10^

Dysregulated microRNAs (miRNAs) are highly involved in the initiation and progression of multiple cancers. They function as either proto‐oncogenes or tumour suppressors in vivo by repressing their target mRNAs or reducing their transcription.^11^, ^12^ The dysregulation of miR‐125b is commonly observed in many malignant tumours, such as ovarian,^13^ colon^10^ and breast^14^ tumours. Our previous study revealed that miR‐125b^9^ not only contributes to cell migration but is also regulated by PAR_2_ activation. In view of this evidence, we believe that the molecular characteristics of miR‐125b, which is regulated by PAR_2,_ should be studied in depth to monitor tumour outcomes.

In the present study, we used multiple online tools to analyse the association between PAR_2_ levels and tumour prognosis in multiple cancer. Moreover, PAR_2_ expression and the clinicopathologic stage of cervical squamous cell carcinoma endocervical adenocarcinoma (CESC) were assessed with a tissue microarray (TMA). miR‐125b and its target Grb‐associated binding protein 2 (Gab2), which are strongly linked to PAR_2_‐induced cell migration, are well‐characterized predictors of metastasis in CESC. Most importantly, The Cancer Genome Atlas (TCGA) data sets of CESC analysed by Empower Stats demonstrated the predictive accuracy of PAR_2_ in CESC metastasis. Therefore, the PAR_2_ expression pattern could serve as a risk factor that indicates a poor prognosis for patients with cervical cancer.

## 2 MATERIAL AND METHODS

### 2.1 Cell culture and cell lines

The human colonic epithelial cell line HT‐29, and HCT116 as well as the lung adenocarcinoma cell line A549 were obtained from the American Type Culture Collection (Manassas, VA, USA). The cells were grown in Dulbecco's modified Eagle's medium/F12 supplemented with 10% FBS (Gibco, NY, USA). Stably transfected HT29 cells with PAR_2_ knockdown were enriched with puromycin according to a previously described protocol.^7^

### 2.2 Gene Expression Profiling Interactive Analysis (GEPIA), Kaplan‐Meier plotter and Gene Expression Display Server (GEDS) online database

Multiple tumour vs normal differential PAR_2_ expression analysis was performed based on the GEPIA database (http://gepia.cancer-pku.cn), which is a newly developed web‐based tool that provides key interactive and customizable functions based on TCGA and genotype‐tissue expression data.^15^, ^16^, ^17^

The prognostic values of PAR_2_, miR‐125b and Gab2 in tumour patients were evaluated using Kaplan‐Meier plotter (http://kmplot.com/analysis), an open online data set that can be used to assess the effects of 54 675 genes on survival in 21 cancer types.^18^, ^19^

The differential expression of miR‐125b between tumours and corresponding non‐tumour tissues was evaluated using the GEDS database (http://bioinfo.life.hust.edu.cn/web/GEDS/) integrates multiscale gene, mRNA, miRNA and protein expression data from 23 315, 9009 and 9244 samples, respectively, from 40 tissues and 1594 cell lines.^20^

### 2.3 TMA and immunohistochemistry (IHC)

Immunohistochemistry studies of PAR_2_ were performed on CESC samples from a TMA. The TMA was obtained from Outdo Biotech Co., Ltd. (Shanghai, China), including 119 CESC and 35 adjacent tissue specimens (Table S1). The patients undergoing surgery from January 2010 to October 2011 were classified based on the tumour node metastasis (TNM) classification system. All specimens were classified based on the tumour node metastasis (TNM) classification system. The primary antibody used for immunostaining was rabbit anti‐human polyclonal antibody‐PAR_2_ (Abcam Co., Cambridge, MA, USA; 1:100 dilution). The secondary antibody used for immunostaining was two‐step plus^®^ Poly‐HRP Anti‐Mouse/Rabbit IgG Detection System (OriGene, Wuxi, China). The staining results were randomly selected and believed to be representative of the average results in the tumours by two independent experienced pathologists blinded to the clinical data.

### 2.4 Real‐time PCR

The total RNA was isolated from cells using TRIzol reagent (Invitrogen, Carlsbad, CA). After treatment with DNase I, RNA was reverse transcribed into cDNA with a Thermo Scientific Maxima First Strand cDNA Synthesis Kit for mRNA and analysed for miR‐125b detection with a TaqMan™ microRNA Transcription Kit. Real‐time quantitative PCR was carried out on an Applied Bio‐Systems 7500 PCR instrument. PCR data were normalized to those of GAPDH and U6 short hairpin RNA for mRNA and miRNA, respectively.

Primers for mature miRNA and U6 were obtained from GeneCopoeia (GuangZhou Ribobio Co. Ltd., China). Additional primers used in this study were as follows: PAR_2_ ^21^ sense, 5′‐TGA AGA TTG CCT ATC ACA TAC‐3′and antisense (5′‐TGC ATT ATT TTC TGA TTA AGA GCC‐3′); and Gab2^9^ sense (5′‐CGC TGC TA5′‐GAC AAC AGC CGA CTT CAC C‐3′) and antisense (5′‐GCC CAC AAT CAT TTT CCC T‐3′).

### 2.5 Statistical analysis

All statistical analyses were performed using GraphPad Prism 5.0, Empower Stats software (www.empowerstats.com, X&Y solutions, Inc Boston MA) and R (http://www.R-project.org).^22^ The data are presented as the mean ± SD, and a *P* value less than .05 was considered statistically significant.

## 3 RESULTS

### 3.1 PAR_2_ is up‐regulated in multiple types of tumours

**FIGURE 1:**
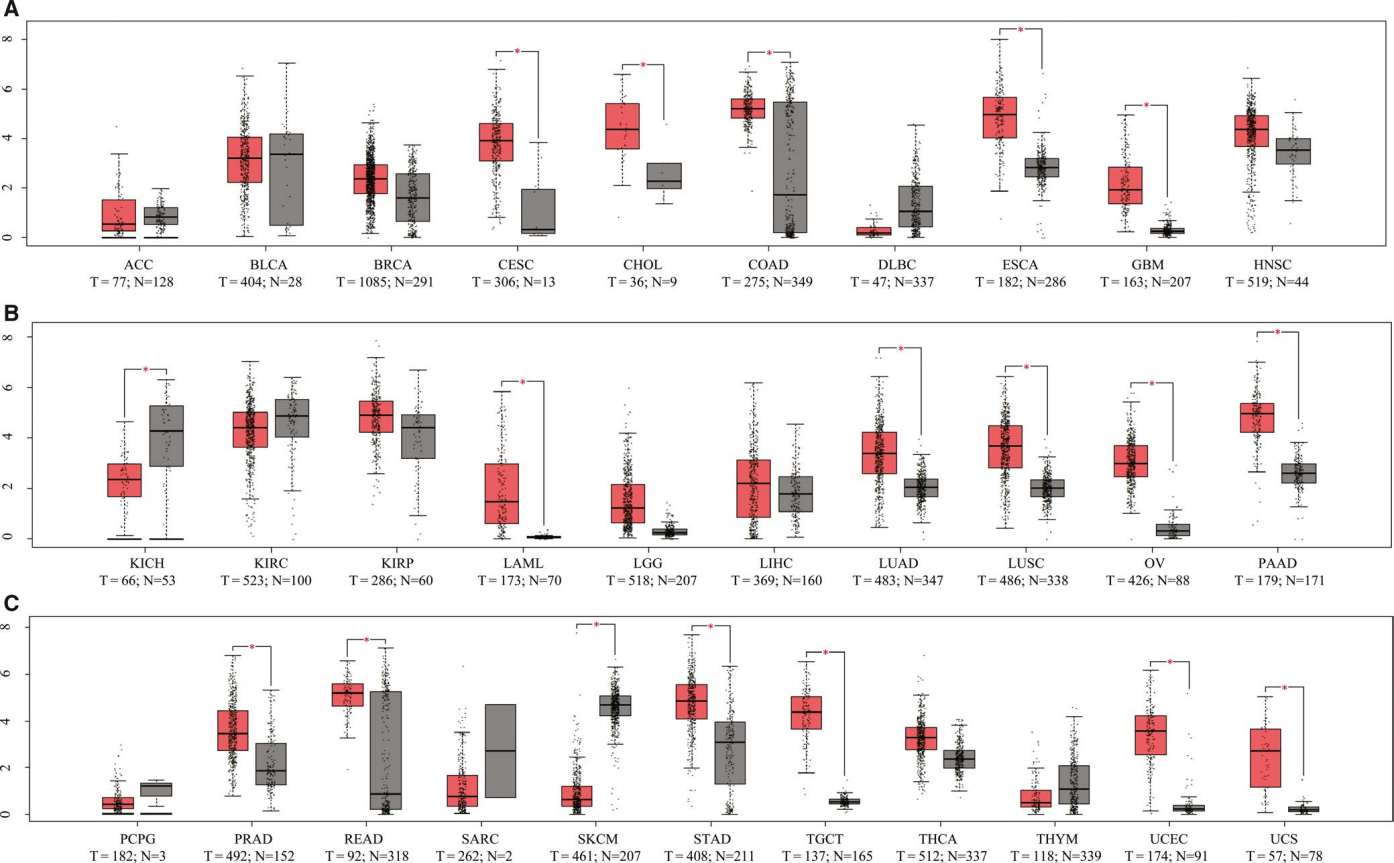
The mRNA expression of PAR_2_ in patients with multiple types of cancer. Differential expression of PAR_2_ in 30 different cancer types. The fold change was calculated as the median expression of PAR_2_ in tumour tissue divided by the median expression of PAR_2_ in adjacent normal tissue. Box plots of PAR_2_ mRNA expression based on GEPIA

We initially found that PAR_2_ was expressed in nearly all human tissues after searching *Gemini* online tools (Figure S1). Moreover, we used the GEPIA online tool to further evaluate whether PAR_2_ expression was different between non‐tumour and tumour tissues in multiple human cancers. Notably, PAR_2_ expression was markedly up‐regulated in tumour tissue relative to control adjacent tissue. As shown in Figure 1, PAR_2_ showed significantly strong up‐regulation in teen types of cancer, including CESC, cholangio carcinoma (CHOL), colon adenocarcinoma (COAD), oesophageal carcinoma (ESCA), glioblastoma multiforme (GBM), acute myeloid leukaemia (LAML), lung adenocarcinoma (LUAD), lung squamous cell carcinoma (LUSC), ovarian serous cystadenocarcinoma (OV), pancreatic adenocarcinoma (PAAD), prostate adenocarcinoma (PRAD), rectal adenocarcinoma (READ), stomach adenocarcinoma (STAD), testicular germ cell tumours (TGCT), uterine corpus endometrial carcinoma (UCEC) and uterine carcinosarcoma (UCS). In contrast, PAR_2_ expression was down‐regulated in kidney chromophobe (KICH). Taken together, these results suggest that PAR_2_ up‐regulation is highly related to the progression of multiple tumours.

### 3.2 PAR_2_ correlates positively with poor survival in CESC, LUAD and PAAD

To identify whether the up‐regulation of PAR_2_ in multiple types of tumours should be employed as an important biomarker for clinical treatment, the impact of PAR_2_ expression on the 5‐year survival rate was evaluated with Kaplan‐Meier plotter. The correlation between PAR_2_ levels and the prognosis of different cancers demonstrated that patients categorized within the PAR_2_ high‐score group had a significantly poor prognosis (Table 1 and Figure S2), especially those with CESC (overall survival (OS) HR = 1.66, 95% CI = 1.01‐2.73, *P* = .0452; relapse‐free survival (RFS) HR = 3.02, 95% CI = 1.26‐7.25, *P* = .009), LUAD (OS HR = 1.87, 95% CI = 1.38‐2.53, *P* = .0000; RFS HR = 1.89, 95% CI = 1.21‐2.95, *P* = .0043), PAAD (OS HR = 2.11, 95% CI = 1.29‐3.45, *P* = .0023; RFS HR = 6.28, 95% CI = 1.8‐21.85, *P* = .0012) and READ (OS HR = 5.16, 95% CI = 1.30‐22.0, *P* = .0112; RFS HR = 0.13, 95% CI = 0.01‐1.10, *P* = .0271). These results confirmed that PAR_2_ expression had an impact on the prognosis (both OS and RFS) of the CESC, LUAD, PAAD and READ cohorts. In the present study, we performed an in‐depth investigation on whether the activation of PAR_2_ was associated with a poor prognosis of CESC.

**TABLE 1:** The prognostic value of PAR_2_ in patients with multiple cancers

Tumour abbr.	OS			RFS		
	HR	95% CI	*P* value	HR	95% CI	*P* value
CESC	1.66	1.01‐2.73	0.0452	3.02	1.26‐7.25	.0091
ESCA	1.91	0.75‐4.83	0.1669	0.36	0.13‐0.99	.0387
LUAD	1.87	1.38‐2.53	0.0000	1.89	1.21‐2.95	.0043
LUSC	1.39	1.02‐1.90	0.0367	0.69	0.40‐1.19	.1843
OV	1.27	0.94‐1.70	0.1243	1.18	0.83‐1.70	.3579
PAAD	2.11	1.29‐3.45	0.0023	6.28	1.8‐21.85	.0012
READ	5.26	1.30‐22.0	0.0112	0.13	0.01‐1.10	.0271
STAD	1.38	0.97‐1.97	0.0732	1.55	0.81‐2.97	.1835
TGCT	3.85	0.35‐42.0	0.2385	2.78	1.2‐6.44	.0127
UCEC	1.51	0.97‐2.33	0.0635	0.84	0.50‐1.43	.5261

Abbreviations: CESC, cervical squamous cell carcinoma and endocervical adenocarcinoma; ESCA, oesophageal carcinoma; LUAD, lung adenocarcinoma; LUSC, lung squamous cell carcinoma; OV, ovarian serous cystadenocarcinoma; PAAD, pancreatic adenocarcinoma; READ, rectal adenocarcinoma; STAD, stomach adenocarcinoma; TGCT, testicular germ cell tumours; UCEC, uterine corpus endometrial carcinoma.

### 3.3 PAR_2_ expression is associated with tumour metastasis

Metastasis is a major cause of death for patients with malignant tumours. To better understand the relevance and fundamental mechanisms of PAR_2_ in tumours, the correlation between PAR_2_ expression and the metastasis characteristics of clinical CESC tumours was assessed.

**FIGURE 2:**
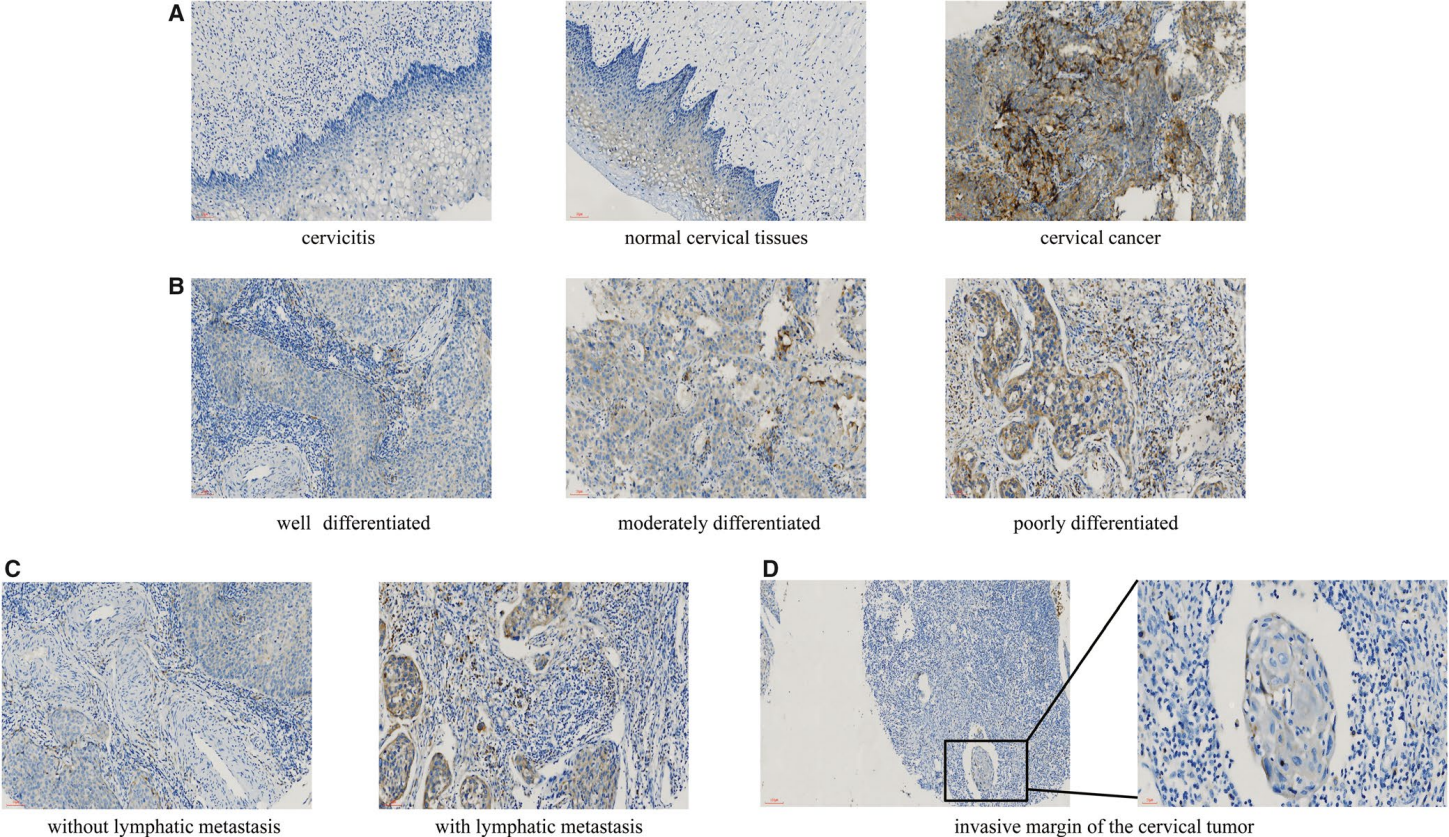
Immunohistochemical staining of PAR_2_ in cervical cancer. A, PAR_2_ was highly expressed in tumour tissue and weakly expressed in normal cervical mucosa and cervicitis tissues (magnification × 200). B, PAR_2_ was significantly up‐regulated in poorly differentiated cervical tumours relative to well‐differentiated or moderately differentiated cervical tumours (magnification × 200). C, PAR_2_ was remarkably up‐regulated in the lymph node metastasis group relative to the nonmetastasis group (magnification × 200). D, PAR_2_‐positive cells observed in front of the invasive margin of tumour tissue (left, magnification × 100); right, magnification × 400)

PAR_2_ expression in 119 CESC patient samples and matched adjacent cervical mucosa samples was assessed with a TMA and IHC staining. The IHC results revealed that PAR_2_ was strongly expressed in CESC tissues, but weakly expressed in normal epithelial tissues of the cervical mucosa and in cervicitis tissues (Figure 2A). Moreover, PAR_2_ expression was more easily observed in poorly differentiated cervical tumours than in moderately or well‐differentiated cervical tumours (Figure 2B). We experienced difficultly in detecting significant differences between tumour stages I and II, but PAR_2_ was up‐regulated in the lymphatic metastasis relative to the local tissue in stage III tumours (Figure 2C). Notably, the PAR_2_‐positive cells were typically stacked in advance of the invasive margin of the tumour tissue (Figure 2D). These results confirm that abnormal PAR_2_ expression is closely associated with the metastasis of CESC.

### 3.4 The levels of miR‐125b and its target Gab2 are closely correlated with a poor prognosis in CESC

Our previous study suggests that miR‐125b mediates PAR_2_‐induced cancer cell migration by regulating Gab2 expression.^9^ To better understand the roles of miR‐125b and Gab2 in clinical CESC progression, the impacts of factors on cell migration were tested. We found that altering the miR‐125b or Gab2 expression changed the cell migration abilities. Notably, the overexpression of miR‐125b by the mimic‐miR‐125b or the knockdown of Gab2 by the siRNA significantly blocked the PAR_2_‐induced cell migration (Figure S3).

**FIGURE 3:**
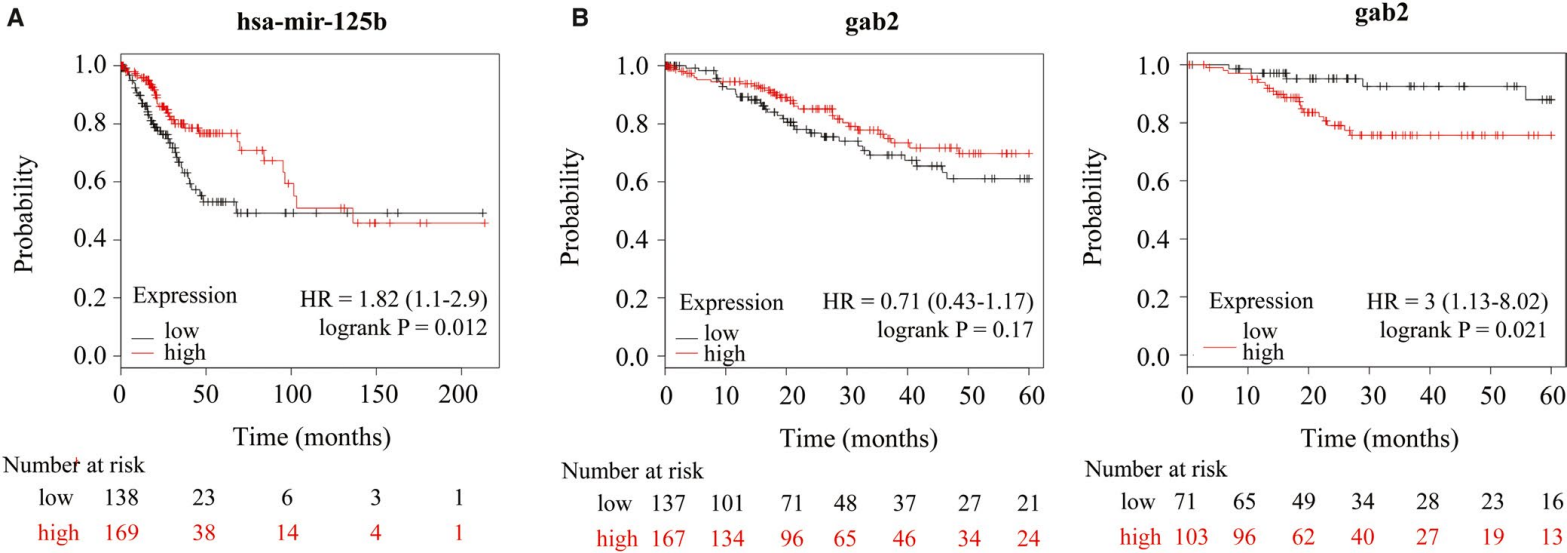
miR‐125b and Gab2 are closely correlated with a poor prognosis in CESC. A, Kaplan‐Meier analysis of the association between miR‐125b and overall survival (OS) in CESC. B, Kaplan‐Meier analysis of the association between Gab2 and OS (left) /relapse‐free survival (RFS) (right) in CESC

Consistent with the findings in vitro, miR‐125b was down‐regulated in multiple types of cancer tissues (Table 2). Moreover, we found that miR‐125b (HR = 1.82, 95% CI = 1.1‐2.9, *P* = .012) expression was significantly associated with poor OS in patients with CESC (Figure 3A). Gab2 was also positively correlated with CESC prognosis (OS HR = 0.71, 95% CI = 0.43‐1.17, *P* = .17; RFS HR = 3.00, 95% CI = 1.13‐8.02, *P* = .021) (Figure 3B). In brief, we combined the Kaplan‐Meier plotter pan‐cancer database (including miRpower^18^ and mRNA) with clinical CESC patient sample data to demonstrate that the PAR_2_‐miR‐125b‐Gab2 pattern serves as a predictive model for prognostic risk in cervical cancer.

**TABLE 2:** The change in miR‐125b expression from adjacent tissue to tumour tissue

Tumour abbr.	Tumour case	Normal case	Tumour expression	Normal expression	Fold change	*P* value
BLCA	408	19	424.66	820.44	0.52	.0002
BRCA	1085	104	723.36	2660.52	0.27	.0000
CHOL	36	9	596.12	1436.06	0.42	.0000
COAD	450	8	257.05	1811.41	0.14	.0000
ESCA	162	11	385.40	443.42	0.87	.6500
HNSC	497	44	470.97	829.92	0.57	.0000
KICH	65	24	1461.43	1012.34	1.44	.8600
KIRC	517	71	467.99	853.99	0.55	.0000
KIRP	289	32	505.76	855.18	0.59	.0000
LIHC	370	50	569.76	1201.99	0.47	.0000
LUAD	512	20	572.02	1249.20	0.46	.0040
LUSC	475	38	585.52	791.90	0.74	.0000
PAAD	178	4	1193.19	926.63	1.29	.2600
PRAD	495	52	1454.75	1371.42	1.06	.8500
STAD	372	32	492.44	617.83	0.80	.0130
THCA	509	58	4268.39	3584.97	1.19	.0520
UCEC	538	33	1062.07	2691.27	0.39	.0000

Note

The differential expression of miR‐125b between tumours/paratumours was analysed by the Encyclopedia of RNA Interactomes (ENCORI) online tool (http://starbase.sysu.edu.cn/panCancer.php).

### 3.5 The prognostic value of PAR_2_ in CESC metastasis

To determine whether PAR_2_ could serve as a prognostic factor for CESC metastasis, the TCGA data set of CESC was downloaded from cBioPortal (https://www.cbioportal.org/datasets, TCGA Pan‐Cancer Atlas) and analysed by Empower Stats software.

**FIGURE 4:**
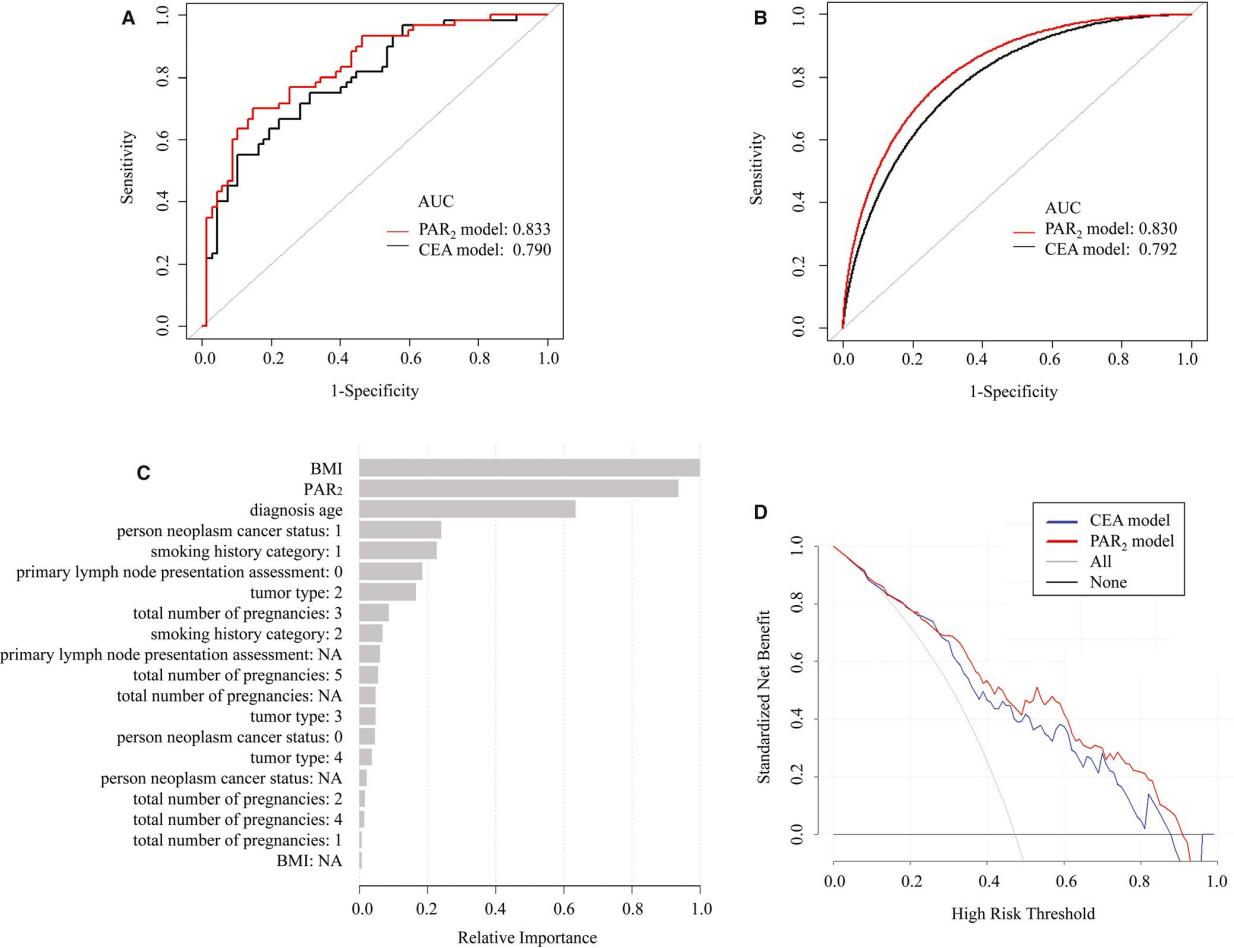
The predictive value of PAR_2_ expression in CESC metastasis. A, Receiver operating characteristic (ROC) curves of the PAR_2_ and CEA model. Area under the curve (AUC) analysis based on the TCGA cohort. B, The bootstrap estimated 95% CI with the area under the ROC curve. C, Importance of the predictive variables in the random forest model, scaled to a maximum of 1. D, Decision curve analysis of the two prognostic models of CESC metastasis. The net benefit curve is shown. Grey line = net benefit when all patients are treated; black line = net benefit when none of the patients are treated. The preferred model is the model with the highest net benefit at any given threshold

The data set contains detailed information on 255 patients (Table 3). Based on the 7th edition of the American Joint Committee on Cancer staging system, we collected recorded items (N = 127) and the risk factors (*P* < .05) that are closely related to tumour metastasis. Then, we formed two predictive models. Data in the CEA model included age at diagnosis, body mass index (BMI), total number of pregnancies, patient smoking history category, tumour type, primary lymph node presentation assessment, neoplasm cancer status and the expression level of CEACAM5, which represents a typical oncofetal antigen.^23^, ^24^, ^25^, ^26^ In the PAR_2_ model, the expression level of PAR_2_ was taken into consideration instead of CEACAM5 (used in the CEA model). Figure 4A shows that the area under the curve (AUC) for the CEA model was 0.790 (95% CI = 0.712‐0.870), yielding a sensitivity of 55.0% and a specificity of 89.6% at the optimal cut‐off value. However, in the PAR_2_ model, the AUC was 0.833 (95% CI = 0.763‐0.903), with a sensitivity of 70.0% and a specificity of 85.1% at the corresponding threshold (*P* = .028). The PAR_2_ model showed a 27.3% (81.6%‐67.3%) increase in sensitivity with comparable specificity at the optimal cut‐off point. As shown in Figure 4B, bootstrap resampling (times = 500) yielded the same result (AUC of the CEA model = 0.792, 95% CI = 0.713‐0.856; specificity = 0.896; sensitivity = 0.550; AUC of the PAR_2_ model = 0.830, 95% CI = 0.765‐0.893, specificity = 0.851, sensitivity = 0.700; *P* = .045). These data demonstrated that PAR_2_ can serve for an important indicator to predict the potential for metastasis in CESC patients.

**TABLE 3:** Basic characteristics of CESC patients with or without metastasis

	American Joint Committee on Cancer metastasis stage code mean ± SD		*P* value
	Without metastasis (N = 114)	With metastasis (N = 141)	
Age at diagnosis (years)	47.8 ± 12.3	48.0 ± 13.7	0.897
BMI (kg/m^2^)	27.6 ± 7.9	27.6 ± 6.6	.971
CEACAM5 mRNA expression level	12 969.53 ± 29 976.7	14 249.3 ± 34 548.7	.756
PAR_2_ mRNA expression level	863.0 ± 685.5	927.6 ± 868.8	.518
	N (%)		
Total number of pregnancies			.986
0	6 (5.9)	7 (5.6)	
1	14 (13.7)	11(8.8)	
2	17 (16.7)	25 (20)	
3	22 (21.6)	23 (18.4)	
4	16 (15.7)	23 (18.4)	
5	11 (10.8)	15 (12.0)	
6	7 (6.9)	9 (7.2)	
7	3 (2.9)	4 (3.2)	
8	1 (1)	1 (0.8)	
9	1 (1)	1 (0.8)	
10	1 (1)	1 (0.8)	
11	1 (1)	3 (2.4)	
12	1 (1)	1 (0.8)	
14	0 (0)	1 (0.8)	
15	1 (1)	0	
Patient smoking history category			.628
1	64 (63.4)	73 (57.5)	
2	19 (18.8)	32 (25.2)	
3	2 (2.0)	5 (3.9)	
4	15 (14.9)	15 (11.8)	
5	1 (1.0)	2 (1.6)	
Tumour type			.051
Adenosquamous	2 (1.8)	4 (2.9)	
Cervical squamous cell carcinoma	95 (87.2)	103 (75.2)	
Endocervical adenocarcinoma	5 (4.6)	13 (9.5)	
Mucinous adenocarcinoma, Endocervical type	2 (1.8)	14 (10.2)	
Endometrioid endometrial adenocarcinoma	2 (1.8)	1 (0.7)	
Usual type	3 (2.8)	2 (1.5)	
Primary lymph node presentation assessment			.074
Yes	83 (87.4)	80 (77.7)	
No	12 (12.6)	23 (22.3)	
Person neoplasm cancer status			
Tumour free	81 (89.0)	81 (67.5)	<.001
With tumour	10 (11.0)	39 (32.5)	

Machine learning methods were used to validate the importance of risk factors in the PAR_2_ model. Figure 4C shows that the PAR_2_ expression level, following the BMI, was the second most important predictor in the random forest model. The decision curve analysis evaluating the benefit and risks of the two models is presented in Figure 4D. The x‐axis and y‐axis show the risk threshold for cancer metastasis and the standardized net benefit using the model, respectively. For the AUC models, the treat all (grey line) and treat none (black line) represent the clinical value for each model. At a relatively large threshold value, the PAR_2_ model was more cost effective than the CEA model. If a threshold of 80% was used as the prediction probability to treat CESC metastasis, then 21/100 patients would benefit from the PAR_2_ model without harming others, compared with 4/100 patients who would benefit from the CEA model.

## 4 DISCUSSION

Cervical cancer ranked fourth in incidence and mortality rates globally among all cancers in women in 2018 (WHO, http://gco.iarc.fr/today/home). Recently, the inspection methods and effective treatment of surgery have continuously improved, which improved the prognosis of CESC. The 5‐year survival rate of CESC patients in the early stage is now over 80.0%, but the appearance of lymphatic metastasis is still one of the main reasons for the difficulty in curing CESC.^27^

Proteases play important roles in the pathological processes of HPV infection,^28^ chronic cervicitis and tumorigenesis. In addition to the proteases produced by inflammatory cells, numerous proteases derived from host cells, and HPV or bacteria are enriched in the uterine cavity. The excessive release of proteases has been reported to be involved in the function and disease states of the cervix. Importantly, some proteases can selectively cleave and activate PAR_2_ signalling.^29^

Previous reports have directly shown that PAR_2‐_induced signalling is essentially related to the migration of cancer cells.^6^, ^9^, ^10^, ^21^, ^30^ PAR_2_ expression is significantly correlated with lymphatic metastasis according to previous reports, but the predictive value of PAR_2_ in tumour metastasis was unrecognized in the past. In the present study, we observed that PAR_2_ expression may be selectively enriched in cancer cells, increasing from the primary local tumour to the corresponding metastatic lymph node lesion and resulting in a poor clinical prognosis.

The EGF receptor (EGFR) has a necessary role in the process of carcinogenesis and is of prognostic and therapeutic relevance in cancer. Some evidence indicates that COX‐2 up‐regulation is dependent on EGFR in cervical cancer,^31^ but activated PAR_2_ can promote EGFR transactivation.^29^ PAR_2_‐induced EGFR activation also up‐regulates tissue factor (TF),^32^ which subsequently activates PAR_2_ to form a feedback loop.

In the recent years, miRNAs have emerged as pivotal regulators in multiple type of cancers. The deregulation of miR‐125b is commonly observed in breast,^33^ ovarian ^34^ and liver^35^ cancers. In the cervix, miR‐125b is up‐regulated in the normal cervical cells infected with HPV, whereas its relative expression becomes down‐regulated as lesions progress.^36^ In our previous report, we confirmed that miR‐125b mediates PAR_2_‐signalling induced cell migration and is closely associated with lymph node metastasis in the colon.^9^ Currently, we showed that OS is short for CESC patients with low miR‐125b expression. Notably, miR‐125b expression is closely regulated by the PAR_2_ status. The continuous activation of PAR_2_ signalling represses the level of miR‐125b, which affects Gab2 expression.

Gab2 is a scaffolding protein that plays an important role in signal integration and amplification.^37^ After its receptors are activated, Gab2 has the ability to interact with Src homology 2 domain‐containing molecules, thereby regulating many biological processes,^38^ which provides the basis for the synergistic action of PAR_2_ signalling. For example, Gab2 acts downstream of EGFR,^39^ which is transactivated by PAR_2_.^40^ However, further studies are needed to determine whether Gab2‐mediated EGFR transactivation occurs through the activation of PAR_2_. Importantly, Gab2 is considered to be a crucial element in the crosstalk and integration of PAR_2_ signalling. Furthermore, in addition to mediating these signalling pathways, Gab2 is also involved in cell migration and tumour progression. Thus, we believe that the importance of Gab2 in tumour progression should also be considered in future investigations.

In summary, we demonstrated that PAR_2_ expression was higher in cervical cancer tissues than in normal tissues and correlated with advanced cancer metastasis and short survival. PAR_2_ activation regulates miR‐125b repression, which may result in its migration‐promoting effect on cancer cells. In addition, our multivariable analysis indicated that PAR_2_ could increase the predictive accuracy of the metastatic prognosis of CESC. We believe that PAR_2_ is an important factor for predicting CESC metastasis, and the change in its expression level should be emphasized in the treatment process of CESC.

## CONFLICT OF INTEREST

The authors declare that they have no competing interests.

## AUTHORS’ CONTRIBUTIONS

HS: Acquisition of data; analysis and interpretation of data; drafting of the paper; and statistical analysis. XM and WL: Acquisition of data; study supervision; and critical revision of the paper. XZ, HY, HQ, LJ and LY: Technical and material support. YL: Study concept and design; obtained funding; drafting of the paper; and study supervision.

## Supporting information

Fig S1

Fig S2

Fig S3

Table S1
