# Supplementary figures and images for: Predictive value of protease‐activated receptor‐2 (PAR2) in cervical cancer metastasis
Source: J Cell Mol Med. 2020 Dec 23;25(3):1415–24. doi: 10.1111/jcmm.16227 (PMC7875903; doi:10.1111/jcmm.16227)

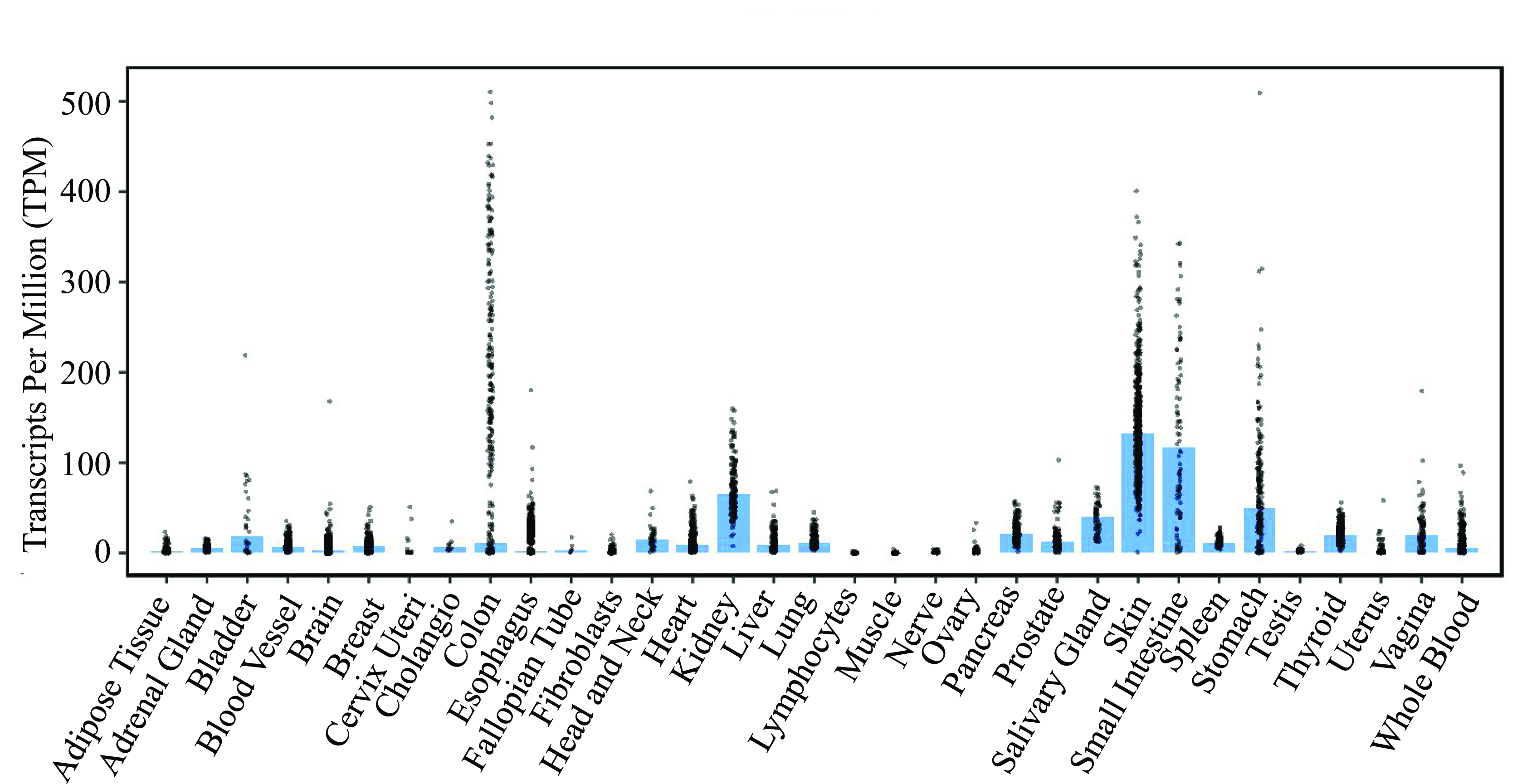

Supplement: Supplementary file 1 — Fig S1 [file JCMM-25-1415-s001.jpg]

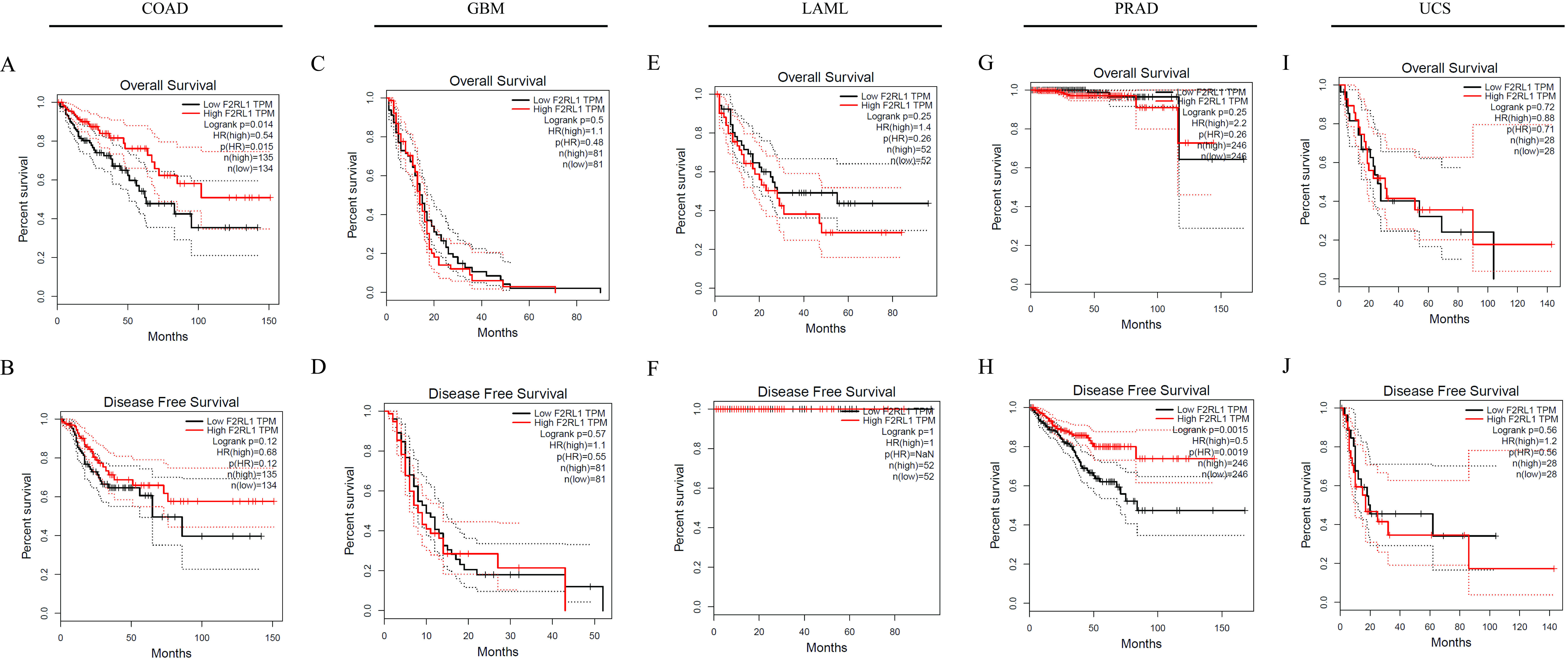

Supplement: Supplementary file 2 — Fig S2 [file JCMM-25-1415-s002.jpg]

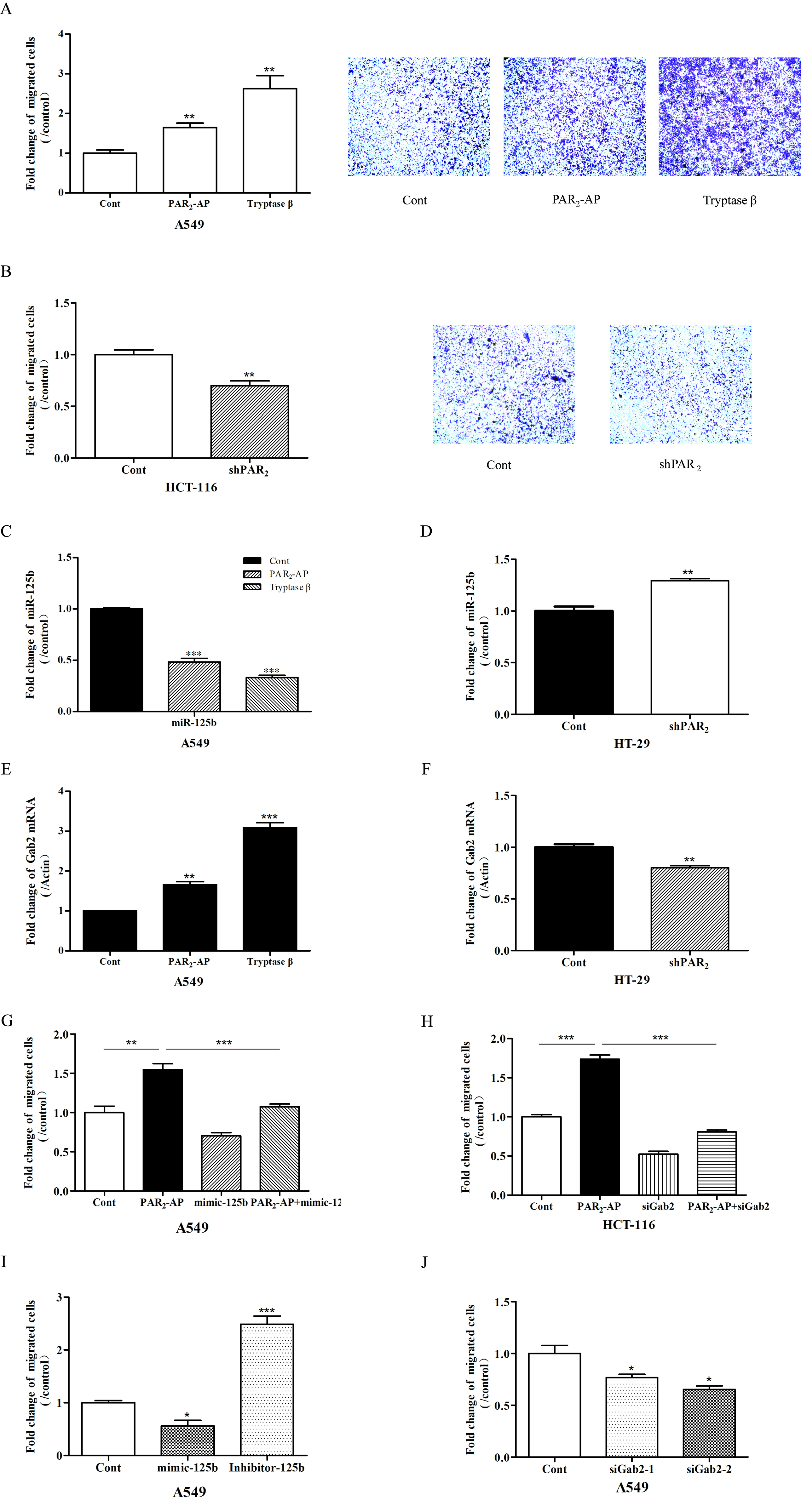

Supplement: Supplementary file 3 — Fig S3 [file JCMM-25-1415-s003.jpg]
